# Study of ^213^Bi and ^211^Pb Recoils Release from ^223^Ra Labelled TiO_2_ Nanoparticles

**DOI:** 10.3390/ma16010343

**Published:** 2022-12-30

**Authors:** Ján Kozempel, Michal Sakmár, Tereza Janská, Martin Vlk

**Affiliations:** Department of Nuclear Chemistry, Faculty of Nuclear Sciences and Pysical Engineering, Czech Technical University in Prague, Břehová 7, 11519 Prague, Czech Republic

**Keywords:** Radium, lead, Bismuth, Ra-223, Bi-213, Pb-211, nuclear recoil, nanoparticles, TiO_2_

## Abstract

Nanoparticles of various materials were proposed as carriers of nuclides in targeted alpha particle therapy to at least partially eliminate the nuclear recoil effect causing the unwanted release of radioactive progeny originating in nuclear decay series of so-called in vivo generators. Here, we report on the study of ^211^Pb and ^211^Bi recoils release from the ^223^Ra surface-labelled TiO_2_ nanoparticles in the concentration range of 0.01–1 mg/mL using two phase separation methods different in their kinetics in order to test the ability of progeny resorption. We have found significant differences between the centrifugation and the dialysis used for labelled NPs separation as well as that the release of ^211^Pb and ^211^Bi from the nanoparticles also depends on the NPs dispersion concentration. These findings support our previously proposed recoils-retaining mechanism of the progeny by their resorption on the NPs surface. At the 24 h time-point, the highest overall released progeny fractions were observed using centrifugation (4.0% and 13.5% for ^211^Pb and ^211^Bi, respectively) at 0.01 mg/mL TiO_2_ concentration. The lowest overall released fractions at the 24 h time-point (1.5% and 2.5% for ^211^Pb and ^211^Bi respectively) were observed using dialysis at 1 mg/mL TiO_2_ concentration. Our findings also indicate that the in vitro stability tests of such radionuclide systems designed to retain recoil-progeny may end up with biased results and particular care needs to be given to in vitro stability test experimental setup to mimic in vivo dynamic conditions. On the other hand, controlled and well-defined progeny release may enhance the alpha-emitter radiation therapy of some tumours.

## 1. Introduction

Alpha-emitting radionuclides have high potential in nuclear medicine. The first modern application of such nuclides, ^223^Ra in targeted alpha therapy (*TAT*), aimed to treat mCRPC in palliative, as well as provide curative treatment of heavily pre-medicated non-responding patients with this disease [1,2]. Radium-223 in the form of ^223^RaCl_2_ was the first alpha-particle cascade emitter approved by FDA and EMA for clinical use for the palliative treatment of metastatic castration resistant prostate cancer [3]. The main differences between the *TAT* and β^-^ particle therapy include the higher linear energy transfer (LET), slightly higher overall energy (5–8 MeV), and very short range in the tissues (50–100 nm) of the alpha particles over the electrons. Even single alpha particle transition through a cell can cause irreparable damage to the DNA double helix, and thus cause cell death [2]. Several other alpha-emitting radionuclides are under clinical investigation, e.g., the ^211^At [4,5], ^227^Th [6], ^225^Ac [7,8,9]. The main pitfall of these cascade alpha particle emitters is the nuclear recoil effect causing the unwanted release and spread of progeny recoils, thus potentially irradiating otherwise healthy and sensitive critical tissues or organs (e.g., salivary glands, kidneys, liver, and spleen) [10].

Nanoparticles and nanomaterials, in general, form a unique platform for drug delivery and advanced systems in nanomedicine [11,12]. This concept could be advantageously transferred to radionuclide therapy, particularly to limit the progeny release [13]. The surface modification of nanoparticles with various organic substances, such as polymers, mAb, and other organic compounds has been used to prevent the natural biodistribution of nanoparticles to the liver or lung and to improve targeting to tumour tissue [14,15]. Many types of nanoparticles have been proposed and tested as carriers for alpha radionuclides in order to least partially prevent the significant release of radioactive progeny originating from the chain decay of used alpha-emitting radionuclides. Studies have included both organic [16,17,18] as well as various inorganic nanoparticles [19,20,21,22,23,24,25,26]. Contrary to the pitfall of the unwanted progeny release, one may benefit from the controlled release of the radioactive nuclei from the nanoparticle or polymer-based brachytherapy carriers, in order to exploit their relocation from their origin by diffusion, convection, or active transport, to irradiate the whole volume of the tumour [27,28,29,30,31,32].

In this study, we have focused on the analysis of progeny release from surface-labelled [^223^Ra]nTiO_2_, depending on the nanoparticle concentration and the method of phase separation of the free progeny atoms from the nanoparticles, to confirm or refute the previously proposed strategy of progeny release elimination by NPs depot formation [13] and possibly evaluate the progeny release in model case conditions.

## 2. Experimental

### 2.1. Study Design

We have proposed three theorems that should be applied in order to minimize the spread of progeny recoils release from their nanocarriers. The recoils spread mitigation could be achieved: (1) by selection of proper time and nuclide (its decay properties), (2) by nanoconstruct size/material, and (3) by the nanoconstructs number/depot size. In this study, we have focused on the mitigation of progeny spread by the nanoconstructs number (or the NPs depot size). In this way, we have studied the influence of NPs dispersion concentration in the range of 0.01–1 mg/mL and the use of two phase separation methods different in terms of phase separation kinetics (centrifugation vs. dialysis) in order to variate the kinetics of the progeny resorption/washout from the NPs depot. Data were collected at five time points in the range of 30 min–24 h. We expected to observe increased ^211^Pb and ^211^Bi progeny release with lower NPs dispersion concentration and higher progeny washout with faster phase separation method.

### 2.2. TiO_2_ NPs Preparation and Radiolabelling

Nanoparticles of TiO_2_ were prepared according to Kukleva et al. [33]. Briefly, a 1:4 mixture of *tert*-butyl orthotitanate with isopropanol was poured dropwise into a distilled water bath under sonication (Branson 450 digital sonicator at 30% power amplitude). Such prepared nanoparticles were washed with deionized water and isopropyl alcohol and dried under vacuum. Nanoparticulate powder was then examined with FT-IR (Nicolet iS50; ThermoScientific, Waltham, MA, USA), XRPD (Miniflex; Rigaku, Tokyo, Japan), and TEM (Tecnai G2 Spirit Twin 12; FEI Company, Brno, Czech Republic). Radiolabelling was performed by the surface sorption of ^223^Ra on the prepared nTiO_2_ according to Suchánková [34]. Firstly, the NPs dispersions in a physiological saline (0.9% NaCl in deionised water) were prepared. Then, ^223^RaCl_2_ (Bayer Pharma AG) was added to the dispersions and left for 12 h to react at laboratory temperature on a shaker. Radium stock solution was not further purified nor modified, and contained water for injections, sodium citrate, sodium chloride, and dilute hydrochloric acid to ensure the isotonicity and pH of the solution in the range of 6–8. Radiolabelled NPs samples were then centrifuged, washed with saline to remove any free ^223^Ra and its progeny, and re-dispersed in saline. Labelling yields exceeded 97% in all cases. Finally, the dispersions of [^223^Ra]TiO_2_ NPs in 3 mL of saline, containing 100–200 kBq of ^223^Ra at 1 mg/mL, 100 µg/mL, 50 µg/mL, 25 µg/mL and 10 µg/mL concentrations were prepared and left to establish the radioactive equilibrium between ^223^Ra and its progeny.

### 2.3. Activity Measurements

All activity measurements were performed on an energy and efficiency calibrated HPGe gamma spectrometer (Ortec, Oak Ridge, TN, USA) with a resolution of 1.85 keV @ 1.33 MeV, using standard calibration sources of ^241^Am, ^133^Ba, ^137^Cs and ^152^Eu (Eurostandard, Prague, Czech Republic). Peaks of 154 keV, 351 keV, 401 keV, and 823 keV were recorded for ^223^Ra, ^211^Bi, ^219^Rn, and ^211^Pb respectively. Stock ^223^Ra samples were measured using calibrated ionization chamber (PTW Curiementor; PTW, Freiburg, Germany). Activities were measured as relative values in defined time intervals to simplify the decay corrections and the same geometry was kept for the sample measurements to keep the constant detection efficiencies. Activities were corrected for decay with a reference time-point at the beginning of phase separation.

### 2.4. Phase Separation Methods

Two different phase separation methods were selected to test the concept of progeny recoils re-implantation or resorption on the NPs surface: dialysis and centrifugation. These methods differ in the separation kinetics, where the dialysis is driven by the diffusion of ions following the concentration gradient while the centrifugation was accelerated by employing centrifugation force.

Centrifugation was performed using the membrane filtration devices (Microsep Advance Centrifugal Devices, Pall, 3 kDa MWCO, Avantor, Stříbrná Skalice, Czech Republic). Labelled NPs dispersion samples of 3 mL were centrifuged in defined time intervals (30, 90, 180, 360 and 1440 min) at 2600× *g* (MPW-360, Warsaw, Poland). An aliquot of 2 mL of the filtrate was then analysed on a gamma spectrometer (Ortec, Oak Ridge, TN, USA). The time between the aliquot sampling and the measurement start was maintained at 4 min (approximately 2 half-lives of ^211^Bi).

Dialysis devices (Pur-A-Lyzer™, 3.5 kDa MWCO; Sigma-Aldrich, Prague, Czech Republic) were used. NPs dispersion samples of 3 mL were dialysed against 30 mL of physiological saline placed in a 50 mL plastic vial. The dialysate was mixed using a magnetic stirrer at 300 rpm. In a defined time interval (30, 90, 180, 360 a 1440 min), an aliquot of 3 mL of dialysate was taken and analysed on a gamma spectrometer and returned after measurement. In such a way, the percentage of free ^223^Ra, ^219^Rn, ^211^Bi, and ^211^Pb was determined. Time between the aliquot sampling and the beginning of the measurement was always maintained at 2 min (approximately one half-life of ^211^Bi). All the pipetted volume uncertainties are estimated to be below 1% according to the pipettes volume calibration.

## 3. Results and Discussion

**Figure 1:**
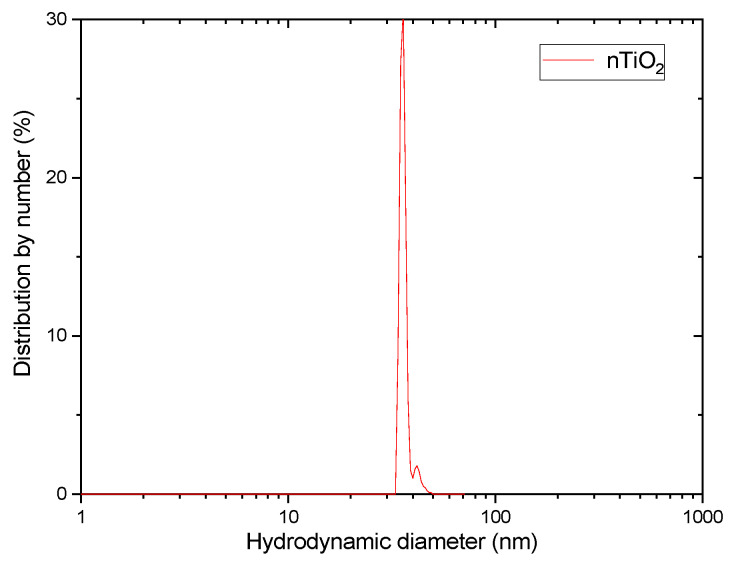
Particle size distribution by number (dilution 1:20).

Full TiO_2_ NPs characterization was performed previously [33]. Analyses performed for the NPs characterization confirmed the anatase phase of the nTiO_2_. Further analyses, e.g., the size distribution and FT-IR, confirmed the composition and their structure. Additional dynamic light scattering (DLS) analysis indicated moderately narrow particle size distribution, having a polydispersity index (PDI) of 0.391; see Figure 1.

**Figure 2:**
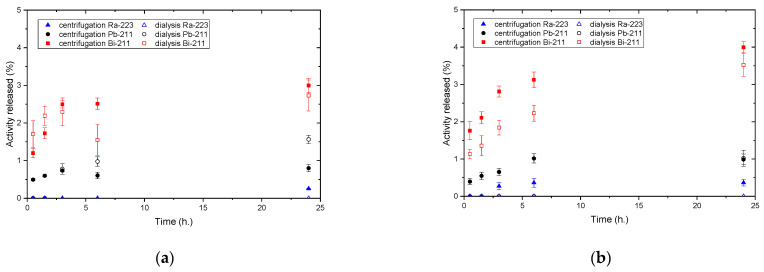

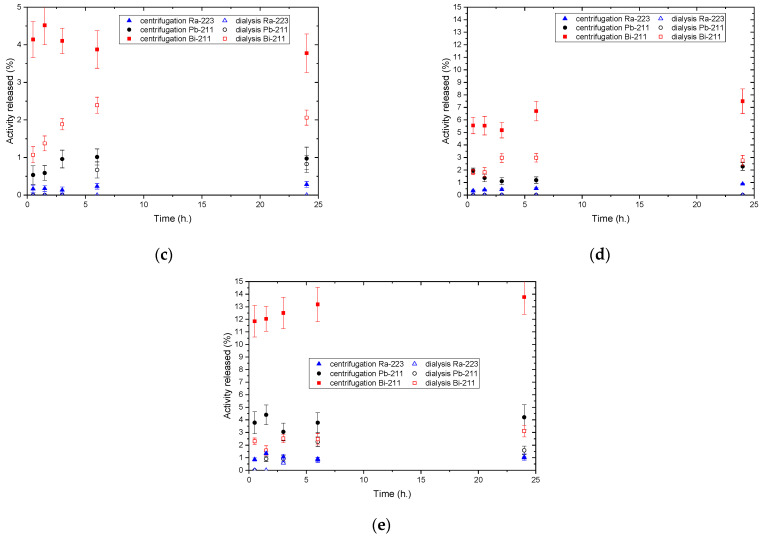
Nuclides release vs. time at (**a**) 1 mg/mL, (**b**) 100 µg/mL, (**c**) 50 µg/mL, (**d**) 25 µg/mL, (**e**) 10 µg/mL dispersion concentration using centrifugation and dialysis as phase separation methods.

**Figure 3:**
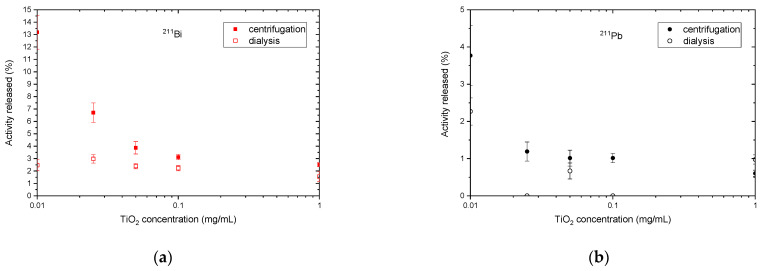
Semi-logaritmic plot of nuclides release vs. nTiO_2_ dispersion concentration for (**a**) ^211^Bi and (**b**) ^211^Pb respectively at 6 h. time-point.

Relatively low ^223^Ra release from the nanoparticles was observed in all tested samples, allowing us to neglect the ^211^Pb and ^211^Bi progeny ingrowth in the separated phases. Time dependencies of activities release of ^223^Ra, ^211^Pb, and ^211^Bi in time from the TiO_2_ nanoparticles are summarized in Figure 2 for centrifugation and dialysis separation methods, respectively, in the range from 1 mg/mL down to 10 µg/mL. It can be clearly seen that the observed progeny release was higher when using centrifugation as the phase separation method compared to slower dialysis. The ^211^Pb and ^211^Bi progeny release dependencies on nanoparticles dispersion concentration at 6 h are shown in detail in Figure 3 for better visibility. It is apparent that the resorption of the studied progeny grows with the dispersion concentrations and thus larger NPs surface available. Further lowering of NPs concentration and larger aggregates together with NPs size lowering and fast phase separation should lead to further progeny release rates as well as total numbers. Both our experiments indicate that the role of resorption of released progeny grows towards approaching static conditions (slow wash-out kinetics, time to approach the equilibrium) and with the NPs available (large sorption area, more stopping mass present in the depot).

## 4. Conclusions

We have previously predicted three strategies for mitigating the nuclear recoil effect and the progeny release from the nanocarriers. Here, we have tested the strategy of possible ^211^Pb and ^211^Bi progeny resorption by a depot of nanoparticles in this study. It could be concluded that the amount of released progeny depends both on the kinetics of phase separation as well as on the NPs concentration in the dispersion or the amount of NPs present in the depot. These effects could be observed thanks to the practically constant generation of ^211^Bi (T_½_ = 2.14 ± 0.02 min.) and ^211^Pb (T_½_ = 36.1 ± 0.02 min.) [35] in the ^223^Ra decay chain where these progeny are in secular equilibrium with ^223^Ra that may be only partially impaired by ^219^Rn emission during the observation time. In our previous work, we have studied the kinetics of ^223^Ra sorption on nTiO_2_ and determined the sorption reaction half-live T_½_ = 0.51 ± 0.32 min. in a closed reaction vial [34]. The same order of magnitude values could be expected for ^211^Pb and ^211^Bi progeny sorption on nTiO_2_. In our previous work, we also came to the assumption that the probability of back-implantation of the first-decay progeny should be statistically close to the value of 50% in the case of surface labelled NPs [19]. This effect should be further supported by the progeny resorption on the NPs surface, if ejected out of the NPs. Holzwarth et al. recently described a good model for progeny release from spherical intrinsically labelled NPs [36].

A comparison of the two phase separation methods showed statistically significant differences in the amount of the released progeny from the labelled NPs in some cases, particularly at low NPs dispersion concentrations (see Figure 2). While the lower values of progeny activities released during relatively slower dialysis indicate a strong effect of the recoils resorption, it is at least partially suppressed in faster separation by centrifugation.

In the second part of our study, we observed an influence of TiO_2_ NPs dispersion concentration on the amount of released progeny. Lowering the dispersion concentration, we have observed expected increase in the recoils release from the NPs phase for both separation methods (see Figure 3). It could be simply deducted that, lowering the NPs available surface for the progeny resorption, a higher progeny release could be foreseen.

Combining the conditions of these model systems (sorption kinetics, open reaction systems, NPs dispersion concentration, etc.) and the observed results, it could be predicted that under real conditions of some in vivo or in vitro experiments, false-positive results of radiolabelled NPs constructs stabilities and stability tests may be observed and the whole tests become compromised.

To summarize, we came to the conclusion that the effect of resorption of daughter progeny was confirmed in our study. Our experiments have strong implications for radiolabelled nanomaterials, particularly in targeted alpha therapy (*TAT*), where the mother nuclei typically decay in a cascade of several consecutive emissions. In vivo stability studies of these nanomaterials designed to retain at least partially the recoiling progeny may bring significantly weaker results than expected from in vitro tests, particularly under static conditions. Thus, the experimental setup of in vitro stability tests should be designed to mimic in vivo dynamic conditions or additional in vivo tests should be performed. On the other hand, controlled progeny release, if used properly, may enhance the alpha-emitter radiation therapy of some larger tumours or resection cavities. The quality control methods, in general, should also reflect the nuclear recoil effect in *TAT* [37].

We understand that the process of progeny release is more complex than discussed in this work. Nevertheless, we are aware of further parameters, such as competing sorption reactions, convection, ions complexation, etc., that may influence the progeny resorption efficiency. Deeper analysis and modelling of this phenomenon would definitely be of general interest. Nanoconstruct stability in *TAT* would very likely always require confirmation by in vivo animal or some advanced dynamic in vitro model test to determine the retention efficiency of radiolabelled nanomaterials under real or very similar to real conditions.

## Data Availability

The data presented in this study are available on request from the corresponding author. The data are not publicly available due to their partial proprietary nature.
